# Evaluation of the colorectal cancer screening Programme in the Basque Country (Spain) and its effectiveness based on the Miscan-colon model

**DOI:** 10.1186/s12889-017-4639-3

**Published:** 2017-08-01

**Authors:** I. Idigoras, A. Arrospide, I. Portillo, E. Arana-Arri, L. Martínez-Indart, J. Mar, H. J. de Koning, R. Lastra, M. Soto-Gordoa, M. van der Meulen, I. Lansdorp-Vogelaar

**Affiliations:** 1 Basque Country Colorectal Cancer Screening Programme, the Basque Health Service, Gran Vía, 62 – 4°, 48011 Bilbao, Spain; 2 BioCruces Health Research Institute, Barakaldo, Spain; 3 Gipuzkoa Primary Care - Integrated Health Care Organizations Research Unit. Alto Deba Integrated Health Care Organization, Gipuzkoa, Spain; 4 Health Services Research on Chronic Patients Network (REDISSEC), Mondragón, Spain; 5 Biodonostia Health Research Institute, San Sebastian-, Donostia, Spain; 6 Department of Public Health, Erasmus MC, University Medical Center, Rotterdam, The Netherlands; 7 Department of Information Technologies, The Basque Health Service, Vitoria-Gasteiz, Spain

**Keywords:** Colorectal cancer, Early detection of cancer, Incidence, Mortality, Life year lost, Effectiveness, Programme evaluation

## Abstract

**Abstract:**

The population-based Basque Colorectal Cancer (CRC) Screening Programme started in 2009 with a biennial immunochemical quantitative test (FIT) biennial and colonoscopy under sedation in positive cases. The population target of 586,700 residents was from 50 to 69 years old and the total coverage was reached at the beginning of 2014. The aim of our study was to determine possible scenarios in terms of incidence, mortality and reduction of Life-years-Lost (L-y-L) in the medium and long term of CRC.

**Methods:**

Invitations were sent out by the Programme from 2009 to 2014, with combined organizational strategies. Simulation was done by MISCAN-colon (Microsimulation Screening Analysis) over 30 years comparing the results of screening vs no-screening, taking the population-based Cancer Registry into account. Lifetime population and real data from the Programme were used from 2008 to 2012. The model was run differentially for men and women.

**Results:**

924,416 invitations were sent out from 2009 to 2014. The average participation rate was 68.4%, CRC detection rate was 3.4% and the Advanced Adenoma detection rate was 24.0‰, with differences observed in sex and age. Future scenarios showed a higher decrease of incidence (17.2% vs 14.7%), mortality (28.1% vs 22.4%) and L-y-L (22.6% vs 18.4%) in men than women in 2030.

**Conclusions:**

The Basque Country CRC Programme results are aligned to its strategy and comparable to other programmes. MISCAN model was found to be a useful tool to predict the benefits of the programme in the future. The effectiveness of the Programme has not been formally established as case control studies are required to determine long term benefits from the screening strategy.

## Background

Colorectal cancer (CRC) is the third leading cancer-related cause of death in developed countries. The European Union (EU) has the highest incident rate and ranks second in mortality of both sexs, with 446,000 newly-diagnosed cases each year and a mortality rate estimated in 214,000 cases annually [1].

In the Basque Country, one of the 17 autonomous regions of Spain, it is also the most frequent type of cancer. In 2008, 642 new cases and 286 deaths in women and 1227 new cases and 504 deaths in men were registered [2].

Different screening strategies have been proposed to reduce the CRC incidence and mortality, by means of different diagnostic tests. Previously, evidence of the reduction in mortality using the guaiac test (gFOBT) for population-based screening, showed a reduction in mortality of 10–16% [3–5].

Although there are few studies demonstrating the impact on mortality of a CRC screening programme using immunochemical quantitative tests (FIT), several clinical trials show that these tests achieve a higher neoplasia detection rate and higher positive predictive values (PPV) than the gFOBT [6–8]. In fact, the European guidelines of screening for CRC (2010) [9] recommended these tests for population-based screening programmes.

A recent study published by Zorzi et al. [10] established that the screening programmes based on FIT were associated with a reduction of up to 22% in CRC mortality.

In accordance with the European recommendation (2003) [11] and the National Health System’s strategy against cancer (NHS) [12, 13], in 2008 the Basque Government approved the implementation of a regional population-based screening programme for CRC. The programme was aimed at men and women between 50 and 69 years old, using one sample biennially of FIT and a colonoscopy under sedation as a diagnostic confirmation in positive cases. The programme started in 2009, reaching almost the whole target population (approximately 586,700 people) at the beginning of 2014. The main results found in the first period showed a high participation rate, as well as high adenoma and CRC detection rates [14, 15].

In order to measure the effectiveness of the Programme and its current strategy in comparison to no-screening, the MISCAN-colon tool [16], widely and internationally validated, was chosen.

The objectives of this study were to predict future scenarios and outcomes for the Basque population and to determine the epidemiological benefits of the screening programme in terms of incidence, mortality and years of life lost (L-y-L).

This kind of evaluation could be useful to those countries rolling out screening programmes in order to implement actions and guarantee their continuation.

## Methods

**Fig. 1:**
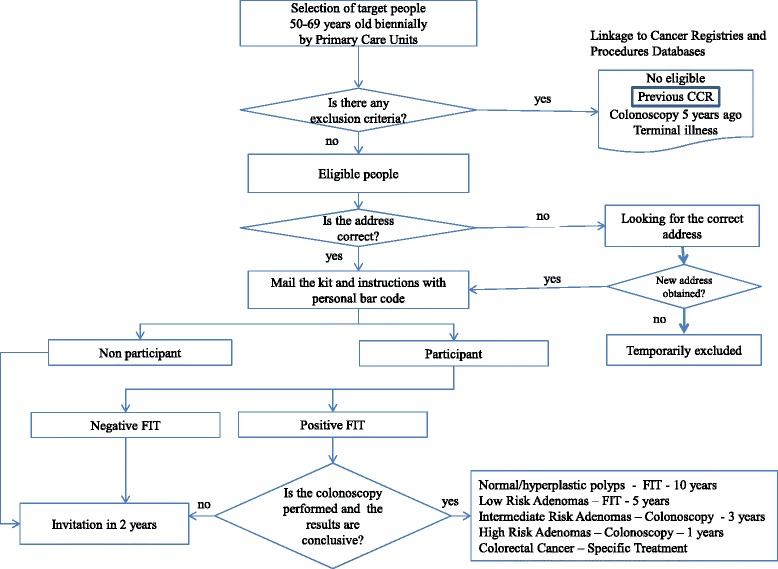
Work flow of the Basque Colorectal Cancer Screening Programme

The Basque Country CRC Screening Programme is population-based and its main strategy was based on: A) a coordinating office, including clinical epidemiologists and statisticians, to plan, organize, manage and evaluate the Programme; B) all residents from 50 to 69 years were invited biennially, taking into account the health centers and referral hospitals, in order to adjust the positivity expected and colonoscopy capacity; C) prior to the invitation, the coordinating office selected the target population and linked the database to the Basque population cancer and medical procedures registries to exclude individuals with a previously-diagnosed CRC, terminal illness and reported colonoscopy in the past five years; D) training and involvement of the primary care staff; E) individualized posted invitations providing information about the Programme. After 4–6 weeks from the initial invitation, the kit (FIT) was sent along with instructions and individualized bar code. This bar code allowed the sample and person to be identified when processing the result. Samples were collected at primary health centers and processed in centralized public laboratories under strict and total quality management systems; F) all results were reviewed by primary care physicians and introduced in “ad hoc” CRC prevention software. Letters were posted with results. In positive cases, participants were recommended to visit their general gractitioner, who referred them to the hospital for colonoscopy. G) Colonoscopies (diagnostic and therapeutic if needed) were performed in public hospitals under deep sedation by specialists. H) All cases were followed-up with close coordination between primary care and specialized units; I) every case was coded by the coordinating office staff following standard EU guidelines and Spanish network consensus [17]; J) interval cancer and complications were identified and monitored by registries linkage before invitation and after colonoscopy performance. The programme is identified in (Fig. 1).

This study was approved by the Basque Country’s Ethics Committee.

The FIT used was OC-Sensor Micro (Eiken Chemical Co. Ltd., Toyo, Japan) (from 2009 until now) and FOB-Gold (Sentinel CH. SpA, Milan, Italy) 2009–2010 in 15,000 invitations). The faecal-Haemoglobin (f-Hb) cut-off was 20 μg Hb/g faeces for both sexs. The decision to use one single sample of FIT and the biennial period between invitations followed the recommendations of Levis and van Rossum [18, 19], in order to reach the highest participation rate with the best balance between sensitivity and specificity.

A satisfactory colonoscopy was considered if the caecum was reached and the quality of colonic cleansing was coded higher than 6 in all segments measured by the Boston Bowel Preparation Scale (BBPS). The American Association’s classification was used for CRC and stages [20]. Accordingly, the results of the colonoscopy were coded and follow-up recommendations assigned to each one as: 1) Normal/No adenomatous pathology and will be invited to perform a screening test within 10 years; 2) Hyperplastic polyps and will be invited to perform a screening test within 10 years; 3) Low risk adenomas and will be invited to perform a test within 5 years 4) Intermediate risk adenomas and remain on colonoscopy surveillance within 3 years; 5) High risk adenomas and remain on colonoscopy surveillance within 1 year; 7) Cancer, neoplasia which infiltrates the submucosa layer ≥pT1) followed by the hospital specialists.

The main results from 2009 to 2014 were used in order to describe the main benefits of the Programme. For the simulation model, the result of the period of 2009–2012 was used for the Basque Country’s inhabitants, and the results obtained from the invitation during 2013–2014 were used to check and contrast the results obtained by the simulation on the MISCAN-colon.

### MISCAN model adaptation

The MISCAN-colon was used to estimate the results of the screening strategy of biennial FIT from 50 to 69 year-olds in the Basque Country. The MISCAN model and the parameter’s sources were fully explained in previous publications [15, 21] and in the standardized model profile of the Cancer Intervention and Screening Network (CISNET) [22]. This model simulates the relevant life histories of a large population of individuals from birth to death. CRC arises in this population in accordance with the adenoma-carcinoma sequence [23].

MISCAN simulated the Basque population in 2008 with its age-structure divided into different strata depending on the age at which they were invited to the Programme for the first time (or never invited if they were over 70 in 2008). Given the significant differences in the epidemiology of CRC between men and women, MISCAN model was run separately for each sex. The validation took into account the stage and localization of CRC in the period of 2005–2008 and the adenoma prevalence calculated for the Basque population using a sample of the COLONPREV study [24].

After reproducing the natural history without screening, the model reproduced the behavior of CRC in a screening scenario by considering the impact of removing adenomas and anticipating CRC stage at diagnosis. Those consequences were translated into quality-adjusted life years gained and treatment costs avoided [25].

In this analysis, the MISCAN-colon model was adjusted to represent the situation of the Basque Country: birth and lifetables and CRC risk and survival from the Cancer Population Register and the Basque Institute of Statistics (EUSTAT) [26]. For the Basque Country, the MISCAN-colon modelling has been adapted to regard the findings of adenomatous lesions, in adenomas smaller and bigger than 10 mm. The projection has been done for 30 years from the implementation of the screening programme. For the prevalence of adenomas, the COLONPREV study and other studies were considered [22, 27, 28].

## Results

### Outcomes of the population-based Basque CRC screening Programme

924,416 individuals were invited (2009–2014), with an average participation rate of 68.4% representing an incremental increase over the study period (58.1% - 70.3%). Trends of participation increased 2.2% yearly (95% CI 2.0–2.4; *p* < 0.001) with 91.8% being regular participants in the second round and 95.8% in the third round. The adherence to colonoscopy after FIT positive result has been higher than 92% in all years of the study. The Advanced Adenoma (AA) detection rate was 23.9‰ and CRC detection rate was 3.4‰. In he 66.4% of CRC cases, the detection was registered in Stage I-II. Indicators by round and sex are detailed in Table 1.

**Table 1:** Main results of the Programme by sex and rounds 2009–2014

	FIRST ROUND				SECOND ROUND				THIRD ROUND				TOTAL				TOTAL	
	WOMEN		MEN		WOMEN		MEN		WOMEN		MEN		WOMEN		MEN			
Eligible People	298,896	%	286,054	%	154,183	%	143,960	%	41,770	%	36,670	%	494,849	%	466,684	%	961,533	%
Invited People	288,775	96.6	273,317	95.5	149,234	96.8	137,705	95.7	40,397	96.7	34,988	95.4	478,406	96.7	446,010	95.6	924,416	96.1
Participants	200,422	69.4	174,968	64	108,776	72.9	93,368	67.8	30,095	74.5	24,427	69.8	339,293	70.9	292,763	65.6	632,056	68.4
Positive test	10,421	5.2	15,671	9	4659	4.3	6422	6.9	1245	4.1	1717	7.0	16,325	4.8	23,810	8.1	40,135	6.3
PPV		%		%		%		%		%		%		%		%		%
Advanced Adenoma		29.4		47.8		23.5		39.0		24.1		40.5		27.3		44.9		37.7
Advanced Neoplasia		34.4		54.5		27.0		43.5		27.5		44.5		31.7		50.8		43.0
CRC		5.0		6.7		3.5		4.5		3.4		3.9		4.4		5.9		5.3
Diagnostic colonoscopy	9720	93.3	14,678	93.7	4282	91.9	5842	91.0	1176	94.4	1595	92.8	15,178	93.0	22,115	92.9	37,293	92.9
Detected Lesions		‰		‰		‰		‰		‰		‰		‰		‰		‰
Advanced Adenoma	3063	15.3	7487	42.8	1094	10.1	2504	26.8	300	9.9	696	28.5	4457	13.1	10,687	36.5	15,144	24.0
Advanced Neoplasia	3583	17.9	8533	48.8	1257	11.6	2796	29.9	342	11.4	764	31.3	5182	15.3	12,093	41.3	17,275	27.3
CRC	520	2.6	1046	6.0	163	1.5	292	3.1	42	1.4	68	2.8	725	2.1	1406	4.8	2131	3.4
CRC Stage		%		%		%		%		%		%		%		%		
I-II	320	61.5	716	68.5	112	68.7	206	70.5	22	52.4	38	55.9	454	62.6	960	68.3	1414	66.4
III-IV	157	30.2	279	26.7	45	27.6	76	26.0	20	47.6	25	36.7	222	30.6	380	27.0	602	28.2
Unknown	43	8.3	51	4.9	6	3.7	10	3.4	0	0	5	7.4	49	6.8	66	4.7	115	5.4

**Fig. 2:**
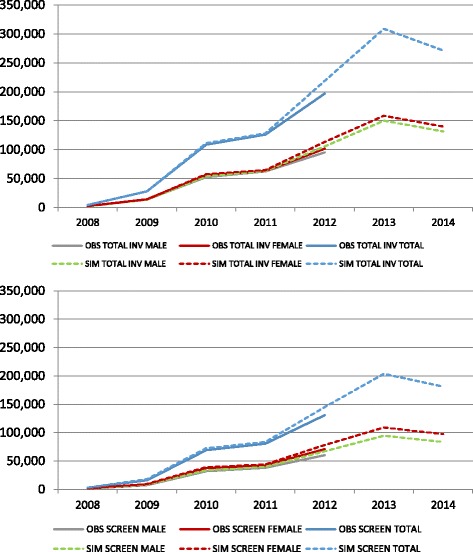
Observed and simulated invitations and participants by sex 2009–2014

Comparing the results obtained on the actual screening scenario with the observed data for invitations, participation rate, positive screen tests and detection rates, we can conclude that the model reproduced well the observed data (Fig. 2).

In Table 2, the future projections were predicted for men and women regarding future invitations, participation, diagnostic/surveillance colonoscopies and detected lesions in different years, the last projection being done in 2038. Observed differences between men and women were noticed in participation, as well as in detected lesions. A trend towards stabilization was observed in all parameters of the projection for 2020 and onwards, but the surveillance colonoscopies seemed to stabilize ten years later.

**Table 2:** Future projections by sex: invitations, participants and lesions detected

	INVITATIONS AND PARTICIPANTS								
	Men population			Women population			Total population		
Year	Invitations	Participants	%	Invitations	Participants	%	Invitations	Participants	%
2012	93,822	58,994	62.9	99,099	68,045	68.7	192,921	127,039	65.9
2015	130,983	85,337	65.2	139,006	98,553	70.9	269,989	183,889	68.1
2020	137,436	91,543	66.6	144,522	104,610	72.4	281,958	196,153	69.6
2025	132,995	88,238	66.3	137,473	99,219	72.2	270,468	187,457	69.3
2030	133,678	88,969	66.6	137,485	99,935	72.7	271,164	188,904	69.7
2035	123,789	83,234	67.2	127,178	93,397	73.4	250,966	176,630	70.4
2038	114,699	77,577	67.6	118,248	87,204	73.7	232,947	164,781	70.7
	DIGANOSTIC COLONOSCOPIES AND LESIONS DETECTED								
	Men population			Women population			Total population		
Year	Colonos copies	Adenomas Detected	CRC Detected	Colonos-copies	Adenomas Detected	CRC Detected	Colonoscopies	Adenomas Detected	CRC Detected
2012	4070	2658	295	2786	1231	142	6856	3888	437
2015	5580	3620	347	3973	1730	185	9553	5350	532
2020	5764	3638	348	4120	1745	159	9884	5384	508
2025	5397	3372	304	3839	1597	148	9236	4969	452
2030	5394	3354	310	3839	1587	148	9233	4941	458
2035	5073	3199	288	3627	1505	140	8700	4704	428
2038	4766	3041	289	3406	1427	134	8172	4468	424
	SURVEILLANCE COLONOSCOPIES AND LESIONS DETECTED								
	Men population			Women population			Total population		
Year	Colonos copies	Adenomas Detected	CRC Detected	Colonos copies	Adenomas Detected	CRC Detected	Colonos copies	Adenomas Detected	CRC Detected
2012	941	259	2	380	101	1	1321	360	3
2015	1971	459	7	900	185	3	2871	644	10
2020	3801	787	16	1832	338	4	5634	1115	20
2025	5190	1172	25	2511	523	9	7701	1695	34
2030	5757	1310	27	2743	571	8	8500	1881	35
2035	5666	1278	25	2706	551	10	8371	1829	35
2038	5523	1233	31	2638	544	9	7561	1777	40
	TOTAL COLONOSCOPIES (Diagnostic and Surveillance) AND LESIONS DETECTED								
	Men population			Women population			Total population		
year	Colonos copies	Adenomas Detected	CRC Detected	Colonos copies	Adenomas Detected	CRC Detected	Colonos copies	Adenomas Detected	CRC Detected
2012	5011	2917	297	3166	1332	143	8177	4248	440
2015	7551	4079	354	4872	1915	188	12,424	5994	542
2020	9566	4425	364	5952	2083	163	15,517	6499	528
2025	10,586	4544	328	6350	2120	157	16,937	6664	485
2030	11,151	4664	337	6582	2158	156	17,733	6822	493
2035	10,738	4477	313	6333	2056	150	17,071	6533	463
2038	10,289	4274	320	6044	1971	143	15,734	6245	463

**Fig. 3:**
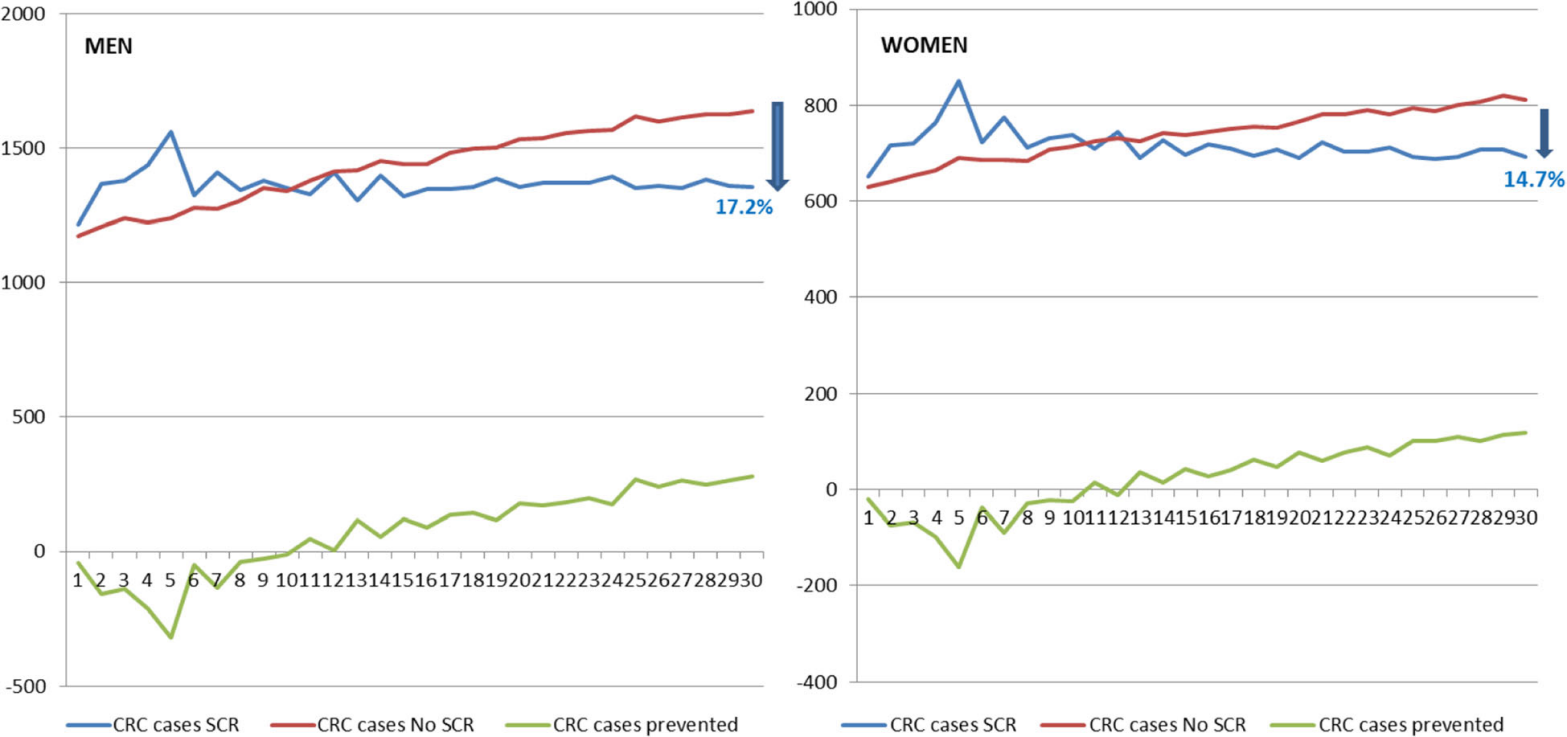
Incidence decreasing for men and women in 30 years

In Fig. 3, a decrease in the CRC incidence was shown after 30 years of screening, greater in men (17.2%) than in women (14.7%). In both sexs, ten years after the Programme started, a decrease was found in the number of cases of CRC. Considering both sexs, the average decrease found was 16.3%.

**Fig. 4:**
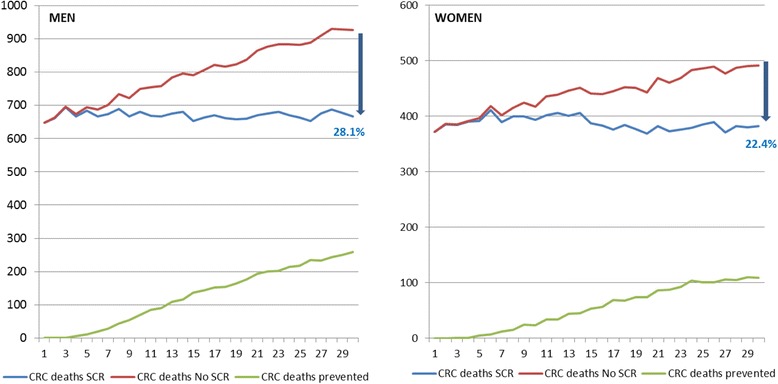
Mortality decreasing by sex in 30 years

Regarding the reduction in mortality for this same projection, the decrease for men was 28.1% and 22.4% for women, with an upward trend from the beginning of the Programme, the average decrease being 26.1% (Fig. 4).

**Fig. 5:**
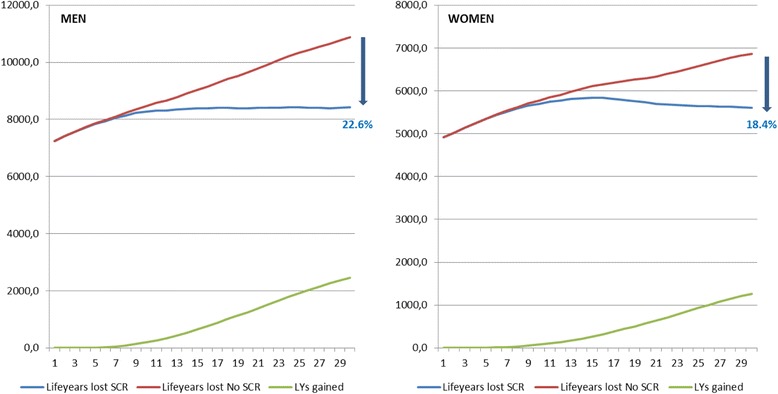
Decreasing in Life years lost by sex in 30 years

The reduction of Life-years-Lost was also greater in men than in women (22.6% vs 18.4%) with an upward trend from the beginning of the Programme and an average for both sexs of 21% (Fig. 5).

## Discussion

The strategy of the CRC Screening Programme in the Basque Country has been implemented according to the recommendations of the EU [9], taking into account the target group and professionals when considering its implementation.

The main results of the Programme showed a high participation rate in both sexs in the three rounds from 2009 to 2014, possibly related to the implemented strategy, according to McGregor et al. [29], who demonstrated a relation to participation in both sexs (men OR 5.0; 95% CI 2.9 to 8.3 and women OR 3.8; 95% CI 2.3 to 6.5). Tinmouth et al. [30] also showed the importance of the family physician when providing information about the programme’s role after Programme invitation. However, Van Roosbroeck et al. [31] demonstrated a higher participation rate related to the type of invitation, higher in shipping kits to a participant’s home than when delivered by the rimary care physician (OR 2.96 95% CI 2.78 to 3.14). Combined strategies could be efficient to achieve a higher participation rate. Also, quality assurance plays an important role (Von Karsa et al., 2013) [32].

The f-Hb cut-off point chosen in FIT has generated a lot of discussion in terms of the number of colonoscopies to be performed, which was an initial limitation to the total extension of the Programme. However, it has not been modified in terms of cut-off age to deal with the management of positive cases, but that should be taken into account in successive rounds, according to van Rossum 2009 [33].

The lesion detection rate analysis reported a high trend in the first round with a significant decrease in successive rounds, following the same pattern as the positive FIT test. The largest decline occurred primarily in men and in AA. Denters et al. [34] found a significant decrease in PPV for AN (Advanced Neoplasia) between the first and the second round of 55% (132/239) to 44% (112/252), (*p* = 0.017). The PPV for CRC was 8% (20/239) in the first round vs 4% (9/252) in the second round (*p* = 0.024).

CRC detected by screening were in early stages (I-II) in 66.4%, contrasting with previous data (45.8%) (Departamento de Sanidad y Consumo et al., 2010) [2].

In the Basque Country Programme, considering its rapid extension and its high participation rate and lesions detected, a positive medium-to -term impact could be expected. This impact was suggested by Zorzi et al. [10] who found a better impact related to geographic locality and the implementation of screening, with higher reductions in mortality in women (RR=0.64; 95% CI = 0.51–0.80) than in men (RR 0.87 95% CI 0.73–1.04), but with significant results in all cases.

The choice of the MISCAN-colon model to simulate the impact of the Programme, both mid and long term, has given us the opportunity to establish a future scenario based on real data, regarding the incidence and mortality before screening as well as the Programme’s results after its start in 2009. One outstanding feature of this method was being able to count on internationally-renowned cancer registers, which make the study of the effectiveness in screening feasible (Anttila et al., 2015) [35].

In this respect, the incidence and mortality rates in the Basque Country are different than in other European regions [36]. When compared with European Population Registers, the Basque Country showed a higher incidence rate in men and an average rate in women compared to the Netherlands, Italy, and Scotland and North Thames in the UK. The mortality rate in men was also higher. However, these incidence and mortality rates showed an intermediate position for women [37].

The simulation applied to sexs offered a wider vision of CRC, which was not reflected in a majority of research, and which was, however, important to calculate the impact of screening programmes. In the current study, the impact of dealing with different population groups was evident, not only regarding the incidence and mortality of CRC, but also how both sexs behaved in participation, positive test rates and the rate of detected lesions. Hence, the programme’s impact was shown to be greater in men than in women, but unfortunately men participated less than women.

After a 30-year projection, and with participation rates adjusted to the results of the Programme, the decrease in incidence and mortality found seems compatible with what is reflected in current literature, although it is difficult to compare results, due to the dissimilarities in context, including simulations of 100% participation and short or indeterminate follow-up periods. However, the quality of simulation and the adaptation of parameters proved successful according to the real data provided by the Programme.

The reduction in incidence would start in the first ten years of the Programme’s implementation with significant increases over time. As other authors have stated, CRC screening not only decreases mortality, but it prevents new cases Ventura et al. [38], which contributes to minimizing the burden of the disease in the future. On the basis of higher incidence rates, the mid-to-long term impact could represent an important reduction in both the number of cases and death, according to Parente et al. [39] who found a significantly lower mortality rate in screening in 5 years compared to non-screening or pre-screening colorectal cancer patients (19% vs 37% and 41%; *p* < 0.001).

In this sense, the L-y-L for both sexs is very high and provided an important tool for regional and national authorities, as well as policy makers, to invest and support these types of programmes, taking into account organization and quality indicators. That recommendation was suggested by van Hees et al., [40] from the Netherlands.

Comparisons between programmes are difficult, as was suggested by Klabunde et al. [41], who found a range of invitation coverage from 30 to 100% and coverage by the screening Programme from 7 to 67.7%, overall participation rate from 7 to 67.7%, and first invitation participation from 7 to 64.3%. These differences could be minimized by implementing different measures to increase coverage and catchment in order to maximize the equality of access and the impact on public health recommended by Senore et al. [42]. One of the limitations of this study is that the classification of the risk of those with removed adenoma, due to the use of MISCAN model, had to be done based only on the size of the lesion, so those identified with other characteristics such as number of adenomas or the grade of dysplasia, had to be proportionally distributed [43].

Another limitation is the uncertainty in estimated adenoma prevalence, which was considerably higher than previously observed in other studies included, to build the MISCAN-colon model. This is, however, consistent with the fact that the study programme has a high participation rate that has been maintained throughout the study period and has not declined in new participants. Based on the robustness of the model, this maintained rate supports the prediction.

An important strength of our study is a well validated model based on several years of data from a high participation-rate population-based programme, directly reported by The Basque Country data and the concordant results observed.

In the future, some findings in FIT performance characteristics, with respect to repeating screening rounds, would be taken into account in order to increase efficiency (van der Meulen et al., 2016) [44]. NowaCurrently, we still have difficulty comparing data from different models, relatedd to a lack of randomized control trials on the effectiveness of FIT, and a lack of data on participation in surveillance and uncertainty in adenoma prevalence. Consequently, there is a need to carry out prospective cohort studies to evaluate the impact of the effectiveness of these programmes within the context of implementation and considering all the possible parameters and their influence.

The results obtained within this research are in line with previously published studies on cost-effectiveness analysis [45].

Nevertheless, the results of the projections offer a rather modest reduction of the main parameters measured. These projections indicate a need to consider how to improve the efficiency of currently implemented strategies. This incudes analyzing the possibility of implementing complementary or improved strategies such as the introduction of algorithms of risks, differentiating among men and women, familiar susceptibility (detected lesions subgroup analysis) or adjusting the cut off levels of the current test. Primary care physicians and authorities are key in maintaining the programme as it is described here. Primary care physicians are central to informing the population about the benefits of being screened and, thus, maintaining the high participation rates. Authorities are important in ensuring the level of investment in order to guarantee that no delays in the subsequent diagnostics and managing processes are generated. The latter is crucial not just from the perspective of the programme itself and its intermediate results (detected lesions as early as possible), but to improve the final outcomes on life expectancy and quality of life.

## Conclusions

The Basque Country CRC Programme results are aligned to its strategy and comparable to other programmes. MISCAN model was found to be a useful tool to predict the benefits of the programme in the future. According to the parameters of simulation of MISCAN-colon and by means of the early obtained data of the Programme, the screening seems to be an effective strategy in order to reduce the incidence, mortality and L-y-L. These results provide further evidence on the efficiency of population-based CRC programmes. These data support the continuity of the programme and show the need for further improvements in the selected strategy to increase its efficiency.

## Abbreviations

AA: Advanced Adenoma
AN: Advanced Neoplasia
BBPS: Boston Bowel Preparation Scale
CISNET: Cancer Intervention and Screening Network
CRC: Colorectal Cancer
EU: The European Union
f-Hb: faecal-Haemoglobin
FIT: Immunochemical quantitative test L-y-L: Life-years-Lost
gFOBT: the guaiac faecal occult blood test
MISCAN-colon: Microsimulation Screening Analysis
PPV: Positive predictive values
